# Beyond the lungs and heart: a possible association between digestive disease and exercise intolerance in 10 dogs

**DOI:** 10.3389/fvets.2026.1915931

**Published:** 2026-10-28

**Authors:** Alexandra Corsaletti, Michaël Martineau, Hugues Etien, Kevin Le Boedec

**Affiliations:** 1 Centre Hospitalier Vétérinaire Frégis-IVC Evidensia France, Service de Médecine Interne, Paris, France; 2 Centre Hospitalier Vétérinaire des Cordeliers-IVC Evidensia France, Service de Médecine Interne, Meaux, France

**Keywords:** aerodigestive disorders, canine, fatigue, gastroenterology, pulmonology

## Abstract

**Introduction:**

Exercise intolerance and panting are classically attributed to cardiac, respiratory, or other diseases. In some cases, the underlying etiology remains undetermined despite a comprehensive diagnostic workup. While fatigue is one of the most common symptoms in humans with inflammatory bowel disease (IBD), chronic enteropathy is not currently recognized as a primary cause of exercise intolerance in dogs.

**Methods:**

A retrospective review was conducted on the medical records of 10 dogs presenting with exercise intolerance lacking overt digestive or respiratory signs. All dogs underwent empirical gastrointestinal-directed medical and dietary therapies with subsequent owner-perceived clinical resolution.

**Results:**

Ten dogs presenting with chronic exercise intolerance were evaluated. Six of these dogs also exhibited unexplained panting at rest (6/10). Subtle gastrointestinal signs and abdominal discomfort on palpation were present in four and five cases, respectively. Standard cardiopulmonary and metabolic investigations were largely unremarkable. Notably, half of the cohort (5/10) exhibited cobalamin insufficiency or deficiency. Dogs were managed with gastrointestinal-directed medical therapies (e.g., prokinetics, acid suppressants, tylosin) and/or dietary modifications (predominantly low-fat, single-protein (monoprotein), or hydrolyzed diets). Following these targeted interventions, all dogs achieved complete clinical resolution per owners within a median of 21 days.

**Conclusion:**

A gastrointestinal etiology may be considered in dogs presenting with unexplained exercise intolerance and excessive panting. Subtle physical exam findings, serum cobalamin and folate concentrations, and empirical dietary/medical trials can be used to support clinical suspicion. This case series suggests a potential canine equivalent of human IBD-related fatigue, highlighting the need for further research into the gut-brain axis, intestinal dysbiosis, and delayed gastric emptying in veterinary medicine.

## 1 Introduction

Fatigue is characterized by weakness or discomfort during physical activity (such as running or walking), leading to exercise intolerance (difficulty or inability to initiate physical activity and/or reduced ability to sustain a physical activity) (1). The exertional threshold that precipitates cessation of activity is variable. In dogs, owners may report early cessation of physical activity, stopping during mild exercise to recover, or generalized fatigue (2). The differential diagnosis for exercise intolerance classically encompasses cardiovascular, respiratory, neuromuscular and metabolic etiology. Cardiovascular causes include arrhythmias, congestive heart failure, thromboembolic disease, and pericardial effusion. Respiratory disorders may contribute to exercise intolerance primarily via hypoxemia, but also through hypercapnia and pulmonary hypertension. Exercise intolerance may likewise be induced by hypoglycemia, uremia, encephalopathies (e.g., hepatic encephalopathy), blood calcium or potassium disturbances, hypothyroidism, and excess or lack of glucocorticoids, whether endogenous or exogenous. Finally, neuromuscular etiology, such as polyneuropathies, polymyopathies, and neuromuscular junction disorders, can also provoke exercise intolerance (3). Obtaining a comprehensive patient history is critical; for instance, the continuous versus episodic character of the clinical signs and some physical examination findings may provide clues to determine the cause of exercise intolerance. For example, a lower motor neuron syndrome involving all four limbs suggests a neuromuscular etiology, whereas identification of *pulsus paradoxus* suggests a cardiovascular cause, most notably pericardial effusion. However, in some cases the cause cannot be determined definitively despite a comprehensive diagnostic workup (4).

With an estimated prevalence of 50–80%, fatigue is one of the most common and persistent symptoms in people with inflammatory bowel disease (IBD), even after remission (5–7). In a study of 250 patients with IBD, 44% reported that their disease limited their ability to exercise, and 82% of these patients attributed this limitation to excessive fatigue (8). The etiology of this fatigue remains incompletely defined and is likely multifactorial in origin: chronic pro-inflammatory state, nutritional deficiency, functional changes in the brain, altered metabolomic profile, psychological factors, and disruption of the gut-brain axis due to dysbiosis (5, 6). Gastrointestinal dysmotility also seems to be linked to fatigue and exercise intolerance (9). In irritable bowel syndrome (IBS), the most common functional gastrointestinal disorder in humans, fatigue has been reported in 9.6 to 98.6% of patients (10).

In veterinary medicine, decreased activity and lethargy are frequently reported in feline and canine chronic enteropathy (CE) (11–13), and two indices commonly used to evaluate canine chronic enteropathy, the CIBDAI (canine inflammatory bowel disease activity index) and the CCECAI (canine chronic enteropathy clinical activity index), both incorporate an attitude/activity component (11, 13). However, exercise intolerance in dogs with chronic enteropathy has not been specifically characterized, and its prevalence remains unknown. Two studies support the possibility that fatigue expressed as exercise intolerance may be an important extraintestinal clinical sign to consider in dogs with digestive disease, though. In horses, one study supported a link between exercise intolerance and equine gastric ulcer syndrome (14). In particular, horses with induced gastric ulceration exhibited reduced time to fatigue and shorter stride length during incremental treadmill exercise testing compared with horses in which ulcer formation was pharmacologically prevented; gastric pain was hypothesized as a potential contributing mechanism (15). In addition, in dogs, exercise intolerance has been reported in some animals with aerodigestive disease. Aerodigestive disorders refer to conditions in which gastrointestinal disorders contribute to respiratory diseases (16). In a retrospective study of 67 dogs with sliding hiatal hernia, exercise intolerance was reported in three dogs, although its relationship with gastroesophageal reflux or aspiration was not specifically assessed (17). Although direct parallels between human and canine gastrointestinal diseases are flawed, fatigue expressed as exercise intolerance appears to be an important extraintestinal clinical sign to consider in dogs with digestive disease.

In this study, we explore a possible association between gastrointestinal involvement and exercise intolerance in a cohort of dogs presenting with exercise intolerance without overt gastrointestinal or respiratory clinical signs, in which gastrointestinal-targeted therapy was followed by owner-reported resolution of excessive panting and exercise intolerance.

## 2 Materials and methods

### 2.1 Inclusion criteria

Dogs presenting with exercise intolerance from 2018 to 2025 were identified in the hospital’s electronic medical records from CHV Frégis, Paris, France. Exercise intolerance was defined as reduced ability to perform or sustain normal physical activity (e.g., walking, running) at the expected intensity or duration. Its presence was determined based on the attending clinician’s assessment of the owner-reported history, as no standardized exercise test was performed. Affected dogs displayed early fatigue expressed as exercise intolerance, with or without excessive panting during exertion. Exertional signs typically improved with rest. Panting at rest was recorded as a secondary finding and was not considered a manifestation of exercise intolerance. Dogs were included in the study if they exhibited exercise intolerance without other respiratory signs (cyanosis, respiratory effort, cough, sneezing, reverse sneezing, or stridor) and without overt digestive signs (vomiting, regurgitation, diarrhea, or anorexia) within 1 month before presentation, and if they responded to medical and/or alimentary therapy directed at chronic gastrointestinal disease or gastroesophageal reflux, excluding the use of corticosteroids. Cases were excluded if they were lost to follow-up, or if concurrent conditions made it difficult to confidently attribute improvements in exercise intolerance to the medical and/or dietary management of gastrointestinal disease.

### 2.2 Diagnostic workup

Dogs were evaluated at presentation and during follow-up by a board certified internal medicine specialist from the American and European Colleges of Internal Medicine (ACVIM[SAIM] and ECVIM-CA) and supervised residents from ECVIM-CA. Body condition score (BCS) was evaluated according to the standard of the Global Nutrition Committee from the World Small Animal Veterinary Association (18). A normal BCS was comprised between 4 and 5 on 9-point scale. Dogs were considered underweight if BCS score was less than 4 and overweight if BCS score was more than 5. Follow-up body weight and BCS were recorded when available; these data were missing for some dogs.

At the clinician’s discretion, ancillary testing was pursued to identify the cause of exercise intolerance in accordance with physical examination findings. The extended diagnostic workup could include: a standard complete blood count (CBC) and plasma biochemistry profile, plasma electrolytes concentrations, plasma ionized calcium concentration, plasma total thyroxine (total T4) and canine thyroid-stimulating hormone concentrations, plasma resting cortisol concentration and adrenocorticotropic hormone stimulation test (ACTHst), plasma creatine kinase concentration, serum folate and cobalamin concentrations, serum DGGR pancreatic lipase activity, fasting and postprandial serum bile acid concentrations, plasma cholesterol and triglycerides concentrations, serum C-reactive protein (CRP) concentration, serum trypsin-like immunoreactivity concentration, arterial blood gas analysis, urinalysis, thoracic radiography, echocardiography and electrocardiogram (ECG), evaluation of the ratio between left atrium and aorta (LA: Ao) using point-of-care ultrasound, thoracic computed tomography (CT) ± angiography, bronchoscopy with bronchoalveolar lavage, and fecal examination with Baermann method. Echocardiographic examinations performed at our hospital were carried out by a board-certified veterinary cardiologist.

Vitamin B12 insufficiency and deficiency were considered based on cobalamin concentrations of less than 400 ng/L and 250 ng/L, respectively.

### 2.3 Data collected

Age, sex, neutered status, breed, clinical signs and their duration, diet history and previous drugs received, and the fat content of the current diet were retrieved from medical records. Heart and respiratory rates, cardiac and respiratory auscultation findings, rectal temperature, and BCS were collected from physical examination reports. Results of all the tests performed by the referring veterinarian and at our hospital were recovered. Finally, drugs and diet changes, diet fat content, and the course of the cases were retrospectively reviewed by the authors.

### 2.4 Statistical analysis

Because of the descriptive nature of the study, no statistical analysis was performed. Continuous data was reported as median value with range values [minimum-maximum].

## 3 Results

### 3.1 Study population

#### 3.1.1 Demographic data

Seventeen dogs were initially identified as having exercise intolerance in the medical record system. However, 1 dog had concomitant myelopathy that necessitated ongoing gabapentin, which confounded attribution of any clinical improvement to gastrointestinal-directed therapy, 1 dog had plasma resting cortisol concentration below the limit to rule out canine hypoadrenocorticism and ACTHst was not pursued, 1 dog had a history of recent severe *Angiostrongylus vasorum* infestation, and 4 dogs were lost to follow-up. Finally, 10 dogs were included in our study.

The population was composed of 6 females, 5 of which were spayed, and 4 males, 3 of which were neutered. The median age at presentation was 5.0 years [1.1–9.9]. Two German shepherd dogs were included; the remaining dogs were each of distinct breeds, including Akita Inu, Bernese mountain dog, border collie, Cairn terrier, Cavalier King Charles spaniel, Flat-Coated retriever, Labrador retriever, and Newfoundland dog. The median weight was 32.0 kg [8.8–66.0] and the median BCS was 5.5 [3.0–8.0]. Three out of 10 dogs had decreased BCS (<4/9) and 5 out of 10 had increased BCS (>5/9). Further details of the included cases are provided in Tables 1, 2. Table 3 summarizes the diets consumed by dogs when information was available and some selected associated analytical constituents.

**Table 1:** Summary of epidemiologic data and history of cases included.

Case	Breed	Age at presentation (year)	Sex and neutered status	Weight (kg)	BCS (/9)	Clinical signs	Duration of exercise intolerance reported by the owners (months)	Drugs administered before presentation
Case 1	Border collie	1.9	FI	14.5	3	Exercise intolerance	24	/
Case 2	Cairn terrier	2.2	FN	10.3	7	Exercise intolerance; unexplained resting panting; excessive yawning; excessive grass-eating	2	Theophylline (9.7 mg/kg PO q12h for approximately 1 month; discontinued 10 days before presentation because panting worsened)
Case 3	German Shepherd dog	6.9	FN	31.0	5	Exercise intolerance; PU/PD; unexplained resting panting; nocturnal arousals with panting; excessive grass-eating; generalized pruritus	36	Omeprazole (administered for at least 1 month before presentation; dosage and administration frequency not recorded)
Case 4	Labrador retriever	5.0	FN	33.0	6	Exercise intolerance; unexplained resting panting	12	Doxycycline (10 mg/kg PO q24h for at least 1 month before presentation); fenbendazole (50 mg/kg PO q24h for 3 days, administered at least 1 month before presentation)
Case 5	German Shepherd dog	1.1	MI	39.0	3	Exercise intolerance	6	Sildenafil (1.25 mg/kg/day for 1 month, approximately 3 months before presentation; no improvement; restarted at 0.5 mg/kg PO q12h 7 days before presentation and continued long-term); fenbendazole (50 mg/kg PO q24h for 5 days, approximately 3 months before presentation; no improvement)
Case 6	Bernese mountain dog	2.1	MN	66.0	8	Exercise intolerance; unexplained resting panting; nocturnal arousals with panting	24	Levothyroxine (18 μg/kg PO q24h for 8 months)
Case 7	Flat-coated retriever	9.9	FN	25.0	4	Exercise intolerance; unexplained resting panting	2	/
Case 8	Cavalier King Charles spaniel	6.7	MN	8.8	6	Exercise intolerance; recurrent throat-clearing episodes; excessive grass-eating	1	Levothyroxine (11.4 μg/kg PO q12h for 2 weeks)
Case 9	Akita Inu	5.2	MN	34.0	3	Exercise intolerance; recurrent throat-clearing; occasional flatulence	0.5	/
Case 10	Newfoundland dog	4.9	FN	55.0	6	Exercise intolerance; rcecurrent throat-clearing episodes; unexplained resting panting; nocturnal arousals with panting; occasional flatulence	5	/

BCS, body condition score; F, female; I, intact; M, male; N, neutered.

**Table 2:** Summary of physical examination findings, diets before treatment, treatments, and follow-up of cases included.

Case	Physical examination findings	Diet before treatment	Treatments	Reported time until clinical signs resolution (days)
Case 1	Hyperthermia; mild abdominal discomfort; underweight (BCS 3/9)	Carnilove Adult Salmon kibble	Diet change; Tylosin powder (dose not recorded; administered with food PO q12h for 30 days)	21
Case 2	Overweight (BCS 7/9)	Home-cooked diet: chicken, rice and green beans	Theophylline discontinued (9.7 mg/kg [100 mg] PO q12h; for approximately 1 month because panting worsened) Famotidine (0.5 mg/kg [5 mg] PO q12h for 3 weeks); Metoclopramide (0.5 mg/kg [5 mg] PO q8h for 3 weeks)	28
Case 3	Grade I/VI right basal diastolic heart murmur; moderate abdominal discomfort; ideal BCS (5/9)	Kibbles: Franklin chicken and sweet potatoes	Diet change; Tylosin powder (¼ teaspoon PO q12h for 30 days)	24
Case 4	Overweight (BCS 6/9)	Kibbles: Crave chicken and turkey	Doxycycline discontinued (10 mg/kg PO q24h; duration before presentation not recorded); Diet change; Metoclopramide (0.3 mg/kg [10 mg] PO q8h for 30 days).	170
Case 5	Underweight (BCS 3/9)	Home-cooked diet: various protein sources	Diet change; Tylosin (¼ dessert spoon PO q12h for 15 days); B12 supplementation (1 mg/dog [2 capsules] PO q24h for 3 months); Sildenafil (0.5 mg/kg PO q12h)	21
Case 6	Hyperthermia; overweight (BCS 8/9)	Kibbles: Pro Plan adult large athletic dog	Levothyroxine discontinued (18 μg/kg [1,200 μg] PO q24h for 8 months); Diet change	30
Case 7	Ideal BCS (4/9)	Wet food: Dog chef chicken or beef	Diet change; Metoclopramide (0.5 mg/kg PO q8h for 1 month, then q12h for 15 days)	12
Case 8	Grade IV/VI left apical systolic heart murmur; moderate abdominal discomfort; overweight (BCS 6/9)	Kibbles: Hill’s metabolic + Hector Kitchen adult	Diet change; Metoclopramide (0.5 mg/kg PO q8h; duration not recorded); Omeprazole (1 mg/kg PO q12h; duration not recorded); Levothyroxine continued (11.4 μg/kg [100 μg] PO q12h)	20
Case 9	Mild abdominal discomfort; underweight (BCS 3/9)	Kibbles: Royal Canin Maxi Adult	Maropitant (2 mg/kg PO q24h for 4 days); Metoclopramide (0.5 mg/kg [15 mg] PO q12h for 1 month, then q24h for 15 days); Sucralfate (3 g PO q8h for 15 days)	16
Case 10	Mild abdominal discomfort; bilateral external otitis; overweight (BCS 6/9)	Kibbles: Select dog gold medical hypoallergenic	Diet change; Maropitant (2 mg/kg PO q24h for 4 days); Metoclopramide (0.5 mg/kg [25 mg] PO q12h for 1 month); Sucralfate (5 g PO q12h for 15 days); B12 supplementation (1 mg/dog [2 capsules] PO q24h for 3 months).	8

**Table 3:** Summary of diets at presentation and during treatment with selected analytical constituents.

Case	Diet at presentation	Diet during treatment	Protein at presentation (g/100 kcal)	Protein during treatment (g/100 kcal)	Total dietary fiber at presentation (g/100 kcal)	Total dietary fiber during treatment (g/100 kcal)	Fat at presentation (g/100 kcal)	Fat during treatment (g/100 kcal)
Case 1	Carnilove adult salmon	Kibbles: Lapsa Maintenance medium chicken	8.7	7.8	NA	NA	4.2	4.4
Case 2	Home-cooked diet: chicken, rice and green beans	No diet change	NA	NA	NA	NA	NA	NA
Case 3	Franklin chicken and sweet potatoes	Kibbles: Purina HA Vegetarian	9.0	5.8	0.9	NA	4.4	2.9
Case 4	Crave chicken and turkey	Kibbles: Hill’s i/d low fat	9.6	7.2	NA	1.6*	4.4	2.4
Case 5	Home-cooked diet: various protein sources	Home-cooked diet: Eggs, pasta and red kidney beans	NA	NA	NA	NA	NA	NA
Case 6	Pro Plan adult large athletic dog	Kibbles: Purina HA Vegetarian	7.0	5.8	NA	NA	4.5	2.9
Case 7	Wet food: Dog chef chicken or beef	Kibbles: Purina HA Vegetarian	NA	5.8	NA	NA	NA	2.9
Case 8	Hill’s metabolic + Hector Kitchen adult	Kibbles: Hill’s i/d low fat	NA	7.2	NA	1.6*	NA	2.4
Case 9	Royal Canin Maxi Adult	No diet change	6.6	6.6	1.6	1.6	4.3	4.3
Case 10	Select dog gold medical hypoallergenic	Kibbles: Hill’s z/d low fat	NA	7.6	NA	2.2	NA	2.4

Analytical values are expressed as g/100 kcal and were calculated from manufacturers’ typical analyses. *For Hill’s i/d Low Fat, total dietary fiber was calculated as the sum of soluble and insoluble fiber reported by the manufacturer. NA, not available.

#### 3.1.2 Clinical signs

All dogs were presented for exercise intolerance, defined in this study as reluctance to walk and lying down during walks or play. Episodes of unexplained panting at rest were reported in six dogs and were accompanied by nocturnal arousals with panting in three dogs. The duration of exercise intolerance varied widely across cases; the median duration was 5.5 months [0.5–36.0]. Clinical signs suggestive of a digestive origin were reported by the owners in 4 out of 10 dogs: recurrent throat-clearing episodes (*n* = 3), excessive grass-eating (*n* = 3), occasional flatulence (*n* = 2), and excessive yawning (*n* = 1). Case 9 was among these dogs and had recurrent throat-clearing episodes and occasional flatulence. One dog had polyuria/polydipsia (PU/PD) for 3 years; previous work-up including CBC, chemistry, abdominal ultrasound, and urinalysis identified no overt cause (case 3). This dog also presented generalized pruritus that partially improved with diet change.

Six dogs received medications before presentation: levothyroxine sodium (cases 6 and 8), theophylline (case 2), omeprazole (case 3), sildenafil and fenbendazole (case 5), and doxycycline and fenbendazole (case 4). Details on these treatment can be found in Table 1 based on available data. Regarding dogs receiving thyroid hormone supplementation, case 6 was previously diagnosed with primary hypothyroidism 8 months prior to presentation, and levothyroxine was initiated in case 8 by the referring veterinarian 2 weeks before presentation because of exercise intolerance. In case 5, a one-month course of sildenafil (1.25 mg/kg/day) and a 5-day course of fenbendazole (50 mg/kg PO q24h), administered approximately 3 months before presentation, failed to improve exercise intolerance. Sildenafil was restarted at 0.5 mg/kg PO q12h 7 days before presentation and was subsequently continued long-term at the same dosage. All prior medications failed to improve exercise intolerance; in case 2, theophylline actually worsened the panting.

#### 3.1.3 Physical examination

Half of the dogs manifested discomfort on abdominal palpation, which was mild in three dogs and moderate in two dogs. Heart murmur was auscultated in two dogs: case 3 had a grade 1/6 right basal diastolic murmur and case 8 had a grade 4/6 left apical systolic one. Respiratory auscultation was unremarkable in all cases. One dog had rectal temperature more than 39.0 °C, most likely attributed to stress. Orthopedic examination was unremarkable in all dogs. Physical examination of each case is summarized in Supplementary Table S1.

### 3.2 Diagnostic workup

Supplementary Table S2a summarizes the diagnostic tests available for the whole study population, and Supplementary Table S2b lists the diagnostic tests performed for each case. Individual values of the various bloodwork performed are presented in Supplementary Table S3. Individual findings supporting suspected gastrointestinal involvement and the corresponding diagnostic interpretation are summarized in Supplementary Table S4.

Nine out of 10 dogs had CBC results available. For case 3, CBC was performed by the referring veterinarian and reported as unremarkable in the medical report. However, the raw laboratory values were not available for review. Only the CBC of case 9 showed an abnormality [mild microcytosis without anemia, presumed to be breed-associated (Akita Inu)]. All dogs had standard biochemistry panels, of which four showed mild abnormalities. Case 4 had moderate isolated increase in ALT activity [ALT = 148 U/L, reference range (RR): 3–69]. Abdominal ultrasonography revealed a diffuse mildly hyperechoic liver and a small amount of biliary sludge, most consistent with vacuolar hepatopathy, especially owing to the high BCS (6/9). Case 7 exhibited mild increase in ALT activity (ALT = 102 U/L) with increased serum CRP concentration (CRP = 31 mg/L, RR: <7) and increased serum cobalamin concentration (vitamin B12 > 1,400 ng/L, RR: 250–900). Additional testing was declined by the owners. Case 9 presented mild increase in plasma urea and creatinine concentrations [urea = 0.60 g/L, RR: (0.16–0.57); creatinine = 17 mg/L, RR: (5–14)]. Abdominal ultrasound and urinalysis were declined by the owner. Finally, case 10 had a mild increase in ALP activity (ALP = 194 U/L, RR: 20–157). Abdominal ultrasound was unremarkable. This case also had a cobalamin insufficiency (vitamin B12 = 343 ng/L), a mildly increased serum CRP concentration (CRP = 14 mg/L), and ACVIM stage B1 myxomatous mitral valve disease (MMVD).

All dogs had serum cobalamin and folate measured. Two out of 10 dogs had mildly increased serum folate concentration (cases 2 and 9) and one has decreased serum folate concentration (case 5). In case 2, abdominal ultrasound revealed focal gastric and diffuse small intestinal mucosal thickening with preserved wall layering, as well as a reduced-size, hyperechoic pancreas, findings suggestive of chronic gastroenteropathy and chronic pancreatitis, respectively. Half of the dogs had abnormally low serum cobalamin concentration, comprising three dogs with cobalamin insufficiency (cases 4, 6 and 10) and two with cobalamin deficiency (cases 3 and 5). Abdominal ultrasound performed in cases 3 and 10 was within normal limits.

Among the seven dogs that underwent thoracic radiography, two showed very mild and diffuse bronchial wall thickening (cases 5 and 6). In case 5, a moderate to severe systolic pulmonary hypertension (gradient: 56 mmHg) was detected by echocardiography performed by the referring veterinarian. A fecal examination using Baermann method was negative. Fenbendazole and sildenafil were prescribed. Echocardiography was repeated at our hospital and confirmed systolic pulmonary hypertension (gradient: 58 mmHg) along with a prominent right ventricle and main pulmonary artery and a small left-to-right interatrial shunt (<1.8 mm) of the sinus venosus type. A bubble test excluded a right-to-left shunt. Thoracic CT angiography was unremarkable. Bronchoscopy coupled with a bronchoalveolar lavage (BAL) was performed. Bronchoscopy revealed a mild erythema of the dorsal mucosa of arytenoids and epiglottis, and a mild and diffuse erythematous bronchial mucosa consistent with bronchitis (Supplementary Video S1). *Mycoplasma* spp. PCR and bacterial culture performed on BAL fluid were negative. The BAL fluid cytological analysis was unremarkable (Supplementary Table S6). However, bile acids were detected in the BAL fluid from the left cranial lobar bronchus (1.1 μmol/L). Bile acid concentration was measured using the same enzymatic colorimetric method routinely used for serum samples. This method has been validated for measuring bile acids within the bronchoalveolar lavage fluid in humans, although sensitivity is inferior to high-resolution mass spectrometry (19).

In Case 6, a mild diffuse bronchial pattern was observed on thoracic radiographs and echocardiography revealed mild aortic insufficiency.

Arterial blood gas analysis was performed in four dogs (cases 1, 3, 4, and 7) and was unremarkable in all of them. Five dogs underwent ECG (cases 1, 3, 4, 7, and 10), all of which were normal.

### 3.3 Treatment and outcomes

Different gastrointestinal-targeted treatments are detailed in Tables 1, 2 and diet is further detailed in Table 3.

Treatments included dietary change (8/10 dogs, 80%; in one dog this was the sole intervention), metoclopramide to enhance lower esophageal sphincter tone and gastric emptying (6/10 dogs, 60%), tylosin to modulate the microbiota (3/10 dogs, 30%), acid suppressants (famotidine or omeprazole; 3/10 dogs, 30%), and gastroprotectant (sucralfate; 2/10 dogs, 20%). Combination of these treatments was frequent (Tables 1, 2). Only two out of the five dogs with cobalamin insufficiency or deficiency received oral vitamin B12 supplementation with Cobalaplex (ADM Protexin Ltd., Somerset, UK) for 3 months. Each capsule contained 0.5 mg of cyanocobalamin, and the daily dose was adjusted according to body weight: 0.25 mg for dogs weighing <10 kg, 0.5 mg for dogs weighing 10–20 kg, and 1 mg for dogs weighing >20 kg. Serum cobalamin concentrations were not reassessed at our hospital after supplementation. Individual baseline cobalamin concentrations, supplementation protocols, timing of reassessment, and time to complete clinical resolution are detailed in Supplementary Table S5.

Case 8, with known hypothyroidism, pursued the same levothyroxine dose given an adequate thyroxine concentration. Thyroid hormone supplementation was stopped in case 6 because of the absence of consistent signs and biological results. For case 5, sildenafil was continued at the same dosage. Theophylline was stopped in case 2, as well as doxycycline and fenbendazole in case 4.

All cases had owner-reported complete resolution of exercise intolerance with a median delay of 21 days [8–170] following treatments and diet change. Complete resolution was defined as an owner-reported return to the dog’s usual activity level, without reluctance to walk or lying down during walks or play, as assessed during follow-up consultations or telephone interviews. No additional medication or treatment changes were recorded in the dog whose complete resolution was reported at day 170 (case 4). Among the six dogs with unexplained resting panting, resolution of panting was reported in five. In the remaining dog, the outcome of panting was not specifically recorded. The subtle digestive signs previously reported also resolved. Interestingly, case 3 had a complete resolution of PU/PD and pruritus with the treatments prescribed (diet change for a low-fat hypoallergenic diet, tylosin, and omeprazole).

Details of dietary composition before and after dietary modification are provided in Table 3.

Changes in body weight and BCS during follow-up are summarized in Table 4. All three initially underweight dogs (cases 1, 5, and 9) were reported to have gained weight and to have improved their BCS. Of the five overweight dogs, cases 2, 4 and 6 remained overweight despite clinical improvement, case 10 decreased its BCS to 5/9, and the change in body weight and BCS was not recorded for case 8.

**Table 4:** Changes in body weight, body condition score, and muscle condition during follow-up.

Case	Weight at presentation (kg)	BCS at presentation (/9)	Muscle condition at presentation	Weight during follow-up	BCS during follow-up	Muscle condition during follow-up
Case 1	14.5	3	Moderate epaxial muscle loss	15.8 kg	Increased to 3.5/9	Improved to mild epaxial muscle loss
Case 2	10.3	7	No muscle loss	10.5 kg	Unchanged (7/9)	Unchanged
Case 3	31.0	5	Moderate epaxial muscle loss	31.0 kg	Unchanged (5/9)	Improved to mild epaxial muscle loss
Case 4	33.0	6	No muscle loss	32.7 kg	Unchanged (6/9)	Unchanged
Case 5	39.0	3	Moderate epaxial muscle loss	NR	Increased to 4/9	Improved to mild epaxial muscle loss
Case 6	66.0	8	No muscle loss	NR	Remained overweight; exact BCS NR	NR
Case 7	25.0	4	No muscle loss	NR	NR	NR
Case 8	8.8	6	Mild epaxial muscle loss	NR	NR	NR
Case 9	34.0	3	Moderate epaxial muscle loss	NR	Increased to 4/9	Improved to mild epaxial muscle loss
Case 10	55.0	6	No muscle loss	NR	Decreased to 5/9	Unchanged

BCS, body condition score; NR, not recorded. Follow-up data include findings documented during clinical re-examinations and information obtained during follow-up.

## 4 Discussion

Although decreased activity and lethargy are frequently reported as nonspecific signs of chronic gastrointestinal disease in dogs, fatigue is not typically considered a core feature of canine chronic enteropathy phenotypes (11, 13). To the best of our knowledge, this case series is the first to report exercise intolerance and excessive panting as the primary presenting complaints in dogs with suspected gastrointestinal disease in the absence of overt digestive signs, such as vomiting and diarrhea. Subtle clinical signs suggestive of a digestive origin, including throat-clearing, grass eating, flatulence, and yawning, were reported in some dogs; however, these signs were not recognized as clinically relevant by owners who initially presented their dogs for respiratory evaluation.

Exercise intolerance and excessive panting can be caused by cardiac, respiratory, or extracardiac/extrarespiratory diseases. In this case series, six dogs underwent comprehensive echocardiographic evaluation, two additional dogs only had left atrium/aorta ratio measurement using point-of-care ultrasound, and an electrocardiogram was performed in five. No relevant cardiac disease was identified to account for the clinical signs. While moderate pulmonary hypertension was detected in one dog, the lack of clinical improvement following sildenafil therapy at 0.5 mg/kg PO q12h does not exclude pulmonary hypertension as a contributor to exercise intolerance and panting, as this dose was lower than doses reported in dogs with pulmonary hypertension (20). Exercise intolerance subsequently resolved without any change in the sildenafil dose. A resolving pulmonary thromboembolism cannot be excluded as an alternative explanation for this improvement, although no thromboembolism was identified on thoracic CT angiography.

A cardiac workup was not performed in the remaining two dogs because a cardiac origin was deemed unlikely by the attending clinician based on the initial clinical evaluation. Respiratory disease can also impair exercise capacity via hypoxia or hypercapnia. To induce fatigue, respiratory compromise should typically manifest with concurrent overt respiratory signs or clinically relevant radiographic abnormalities, neither of which was observed in any dog in this case series. Arterial blood gas analyses were unremarkable in the four dogs evaluated, rendering a respiratory origin for the exercise intolerance and excessive panting highly unlikely in those dogs (21). One dog underwent thoracic CT and bronchoscopy due to concurrent pulmonary hypertension. Although arterial blood gas parameters were not analyzed in this dog, the minor changes noted in the respiratory tract were considered unlikely to account for clinically relevant hypoxemia. This conclusion was further supported by BAL fluid analysis, which revealed no clinically relevant inflammation (total nucleated cell count and differential leukocyte percentages within normal limits). In this context, a link via the gut-lung axis (wherein intestinal dysbiosis may have contributed to the development of pulmonary hypertension) may be considered, although intestinal dysbiosis was not assessed in this dog (22). In people, pulmonary hypertension has been linked to decreased gut microbiome diversity, with reduced production of anti-inflammatory short-chain fatty acids and secondary bile acids and increased production of proinflammatory metabolite trimethylamine by the gut microbiota. These changes are suspected to promote pulmonary vascular disease, contributing to pulmonary hypertension (23). Unfortunately, to the authors’ knowledge, pulmonary hypertension was not reassessed following treatment with antibiotics, cobalamin supplementation, and dietary modification. Although BAL fluid bacterial culture and *Mycoplasma* spp. PCR were negative and BAL cytology was unremarkable, a contribution of the administered antibiotic treatment (tylosin) to the resolution of exercise intolerance cannot be excluded. Sildenafil, which had not improved exercise intolerance before presentation, was continued at the same dosage during gastrointestinal-directed treatment.

When cardiac and respiratory causes were deemed unlikely, most other causes of exercise intolerance and excessive panting were ruled out through physical examination and clinicopathologic and endocrine testing tailored to each dog.

In this case series, a gastrointestinal origin for the exercise intolerance and excessive panting was suspected primarily after the exclusion of other major differentials, alongside the presence of subtle ancillary clinical signs (throat-clearing, grass eating, flatulence, and yawning) and few clinicopathologic changes. Half of the dogs exhibited cobalamin insufficiency or deficiency and two additional dogs had increased folate concentrations, directing clinicians toward an underlying intestinal disorder (24). Interestingly, both dogs with cobalamin deficiency were German shepherd dogs, a breed overrepresented among dogs with low serum cobalamin concentrations and associated with an increased risk of inflammatory bowel disease (25, 26). Consequently, serum cobalamin and folate measurements should be routinely considered in the diagnostic evaluation of dogs presenting with unexplained exercise intolerance and excessive panting. Conversely, while abdominal ultrasonography was performed in five dogs, relevant gastrointestinal abnormalities were detected in only a single one. This highlights that a normal abdominal ultrasound does not exclude chronic enteropathy due to limited sensitivity (27).

Ultimately, the hypothesized digestive origin of these respiratory signs was considered possible based on the favorable clinical response to gastrointestinal-directed medical treatment and dietary modifications.

Interestingly, the subtle digestive signs also resolved concurrently with the exercise intolerance and excessive panting, further supporting a possible association with suspected gastrointestinal disease.

Therefore, these subtle ancillary digestive signs should not be overlooked, as they can raise the index of suspicion for an underlying gastrointestinal origin in cases of unexplained exercise intolerance and panting. Interestingly, a parallel can be drawn to human medicine, where more than 70% of patients referred for idiopathic, life-altering fatigue, and exercise intolerance are diagnosed with some form of gastric dysmotility or discomfort (9). In dogs, such visceral discomfort is a frequently proposed mechanism for these ancillary digestive signs (28).

In humans, fatigue is the most prevalent symptom reported by patients with an inactive state of IBD (7). Although the exact underlying mechanism remains poorly defined, several hypotheses can be proposed that may also apply to dogs (6). Like human IBD, canine CE is associated with a pro-inflammatory state characterized by activation of cytokines such as IL-6 and IFN-γ (6, 29). In human medicine, IBD activity is linked to fatigue, with a higher prevalence observed in patients experiencing active inflammation (6). However, no significant differences in immune markers, such as calprotectin, CRP, and various cytokines, have been demonstrated between patients in remission with or without fatigue, suggesting that inflammation alone may not explain fatigue in quiescent disease (30). In this case series, no dogs showed an inflammatory leukogram but serum CRP was slightly increased in two animals. Unfortunately, serum CRP was not reassessed after exercise intolerance and excessive panting resolved. Anemia and micronutrient deficiencies (such as calcium, iron, niacin, thiamine, folate, cobalamin, and vitamin D) may also contribute to fatigue in IBD patients (6, 30, 31). None of the dogs in this case series displayed anemia but iron-deficiency could be suspected in three animals based on marginally decreased mean corpuscular hemoglobin concentration, mean cell volume, or hemoglobin concentration. However, hematologic parameters can be unreliable markers of iron deficiency in dogs and direct measurement of serum iron, transferrin, ferritin, and total iron binding capacity provides a more accurate assessment (32). These were not evaluated in any of our cases. Five dogs exhibited cobalamin insufficiency or deficiency and two of them received vitamin B12 supplementation; therefore, cobalamin supplementation may have contributed to the resolution of exercise intolerance and excessive panting in these dogs, although its individual effect cannot be determined (33).

Although serum vitamin D concentrations were not evaluated in this case series, vitamin D deficiency is mainly recognized in dogs with protein losing enteropathy or immunosuppressant-responsive enteropathy, which were unlikely in our cohort based on normal serum albumin concentrations and the favorable response to diet change and first-line therapies (34). Furthermore, plasma ionized calcium concentrations were measured in eight dogs and remained within reference intervals, rendering vitamin D deficiency less probable. Two dogs presented with elevated serum folate concentrations, suggesting dysbiosis. Dysbiosis may also have been present in other dogs within this cohort, as concurrent malabsorption can lower folate concentrations, thereby diminishing or normalizing serum values and masking hyperfolatemia (24). In human medicine, fatigue in IBD patients who are in clinical and endoscopical remission has been linked to decreased diversity of the gut microbiota, which also is a hallmark of canine CE (31, 35). Alterations in the intestinal microbiota may contribute to fatigue through changes in microbial metabolites, neurotransmitter signaling, and neuroinflammation (31, 36). Furthermore, gut microbiota dysbiosis is considered a major contributor to myalgic encephalomyelitis/chronic fatigue syndrome (ME/CFS) in humans, a complex disorder characterized by severe fatigue as its core symptom, often accompanied by cognitive impairment, sleep disturbances, musculoskeletal pain, and orthostatic intolerance (37). Although serum cobalamin and folate concentrations were measured in all dogs and provided indirect information regarding intestinal absorption, no microbiome, metabolomic, cytokine, neurotransmitter, or hypothalamic–pituitary–adrenal axis assessments were performed. Therefore, the proposed mechanisms remain speculative.

High rates of sarcopenia and obesity have been reported in human patients with IBD, and both have been linked to fatigue (38, 39). In this case series, body condition score was decreased (i.e., ≤ 3/9) in three dogs and notably increased (i.e., ≥ 7/9) in two. All underweight dogs had improved body condition scores and muscle condition following targeted dietary and therapeutic interventions (Table 4); therefore, disease-associated muscle wasting may have contributed to the exercise intolerance in these animals. Alternatively, muscle gain could also simply reflect successful clinical control of the underlying CE. Conversely, both overweight dogs remained overweight despite clinical improvement (case 2 gained 0.2 kg, and body weight was reported as stable in case 6), suggesting that obesity was unlikely to be the sole contributor to their initial clinical signs. However, exact follow-up weights were unavailable for cases 6 and 8; therefore clinically relevant weight loss cannot be excluded. Abdominal pain resulting from delayed gastric emptying, bloating, gastric hyperacidity, intestinal dysmotility, or gastroesophageal reflux has been suspected to participate in the development of fatigue in IBD patients (1). Interestingly, at initial presentation, half of the dogs in our cohort displayed abdominal discomfort on palpation, which typically resolved alongside the exercise intolerance and excessive panting. Like the previously mentioned ancillary digestive signs, abdominal discomfort upon palpation should not be overlooked by clinicians, as it may serve as an indicator of an occult gastrointestinal disease. Finally, exercise intolerance and excessive panting can result from aerodigestive disorders. Aerodigestive disorders encompass a wide range of conditions characterized by overlapping lesions from gastroesophageal reflux between anatomic regions shared for breathing and swallowing (16). This specific mechanism was suspected in a previous study assessing the prevalence of respiratory signs in dogs with chronic gastroenteropathy, where the majority of the dogs with respiratory signs presented with coughing, vomiting, or gagging (40). Although none of the dogs in our case series exhibited cough, an aerodigestive disorder could be suspected to contribute to exercise intolerance and excessive panting in at least the two dogs that did not undergo dietary modification. Both dogs experienced clinical improvement following treatment with a prokinetic (metoclopramide) and gastroprotectant therapy. Finally, bile acids were detected in the BAL fluid of case 5. While the clinical significance of this finding is unknown, this may suggest reflux in this dog.

All dogs received gastrointestinal-directed medical therapy (prokinetics, acid suppressants, mucosal protectants, or tylosin) or dietary modifications, which resulted in the resolution of exercise intolerance and excessive panting. Eight dogs underwent dietary trials, usually using highly digestible, low-fat, or hydrolyzed diets. The observed clinical improvements may have been associated with the gastrointestinal-directed interventions, although successful control of underlying CE or enhanced gastric emptying could not be confirmed.

Diet alone is effective in the majority of canine CE cases, with highly digestible, low-fat, and hydrolyzed formulations representing the most commonly used dietary strategies (41, 42). Transitioning to a restricted or hydrolyzed protein diet alters gut microbiota composition, reduces dietary antigenicity, and mitigates intestinal inflammation (43). Additionally, six dogs were transitioned to diets intended to reduce dietary fat intake. Standardized before-and-after fat contents were available for three dogs and decreased from 4.4–4.5 to 2.4–2.9 g/100 kcal (Table 3). For the remaining three dogs, complete standardized comparisons were not possible because typical nutrient analysis was unavailable. In human medicine, low-fat, highly digestible, and isoflavone-rich diets accelerate gastric emptying time in patients with gastroparesis (44). Four dogs (cases 3, 6, 7, and 10) were transitioned to soy-based hydrolyzed-protein diets, including a low-fat formulation in case 10. The naturally high isoflavone content of these diets may have contributed to clinical improvement through effects on gastric motility; however, gastric emptying was not assessed, and the contribution of individual dietary components cannot be determined. Two dogs did not undergo dietary modifications but were treated with metoclopramide, in combination with either famotidine or sucralfate. The clinical improvement observed in these dogs may be consistent with the hypothesis that delayed gastric emptying contributed to exercise intolerance and excessive panting, mirroring suspected pathophysiological mechanisms in human patients (45). However, the contribution of enhanced gastric emptying or any individual treatment effect could not be determined.

This clinical observational study has several limitations. Owing to its retrospective design, an exhaustive diagnostic investigation was not pursued in every case; for instance, arterial blood gas analysis was performed in only four dogs, and thoracic CT, bronchoscopy, and BAL in only a single one. Although the assay was analytically validated for bile acid measurement in BAL fluid in humans, it has not been specifically validated in dogs and the clinical significance of the bile acid concentration measured in this single dog remains uncertain. Instead, the diagnostic workup was tailored by the attending clinicians based on the most pertinent hypotheses for each individual dog. Therefore, the extent to which alternative diagnoses were excluded varied among dogs, with some undergoing more extensive cardiopulmonary investigations than others. Consequently, differentials considered highly unlikely were not exhaustively explored. Importantly, no standardized confirmatory gastrointestinal investigations, such as endoscopy with histopathology, assessment of gastric emptying or intestinal motility, or microbiome analysis, were performed, precluding confirmation of the proposed gastrointestinal diagnoses and mechanisms. Furthermore, some dogs had known comorbidities, including previously diagnosed primary hypothyroidism in case 6 and pulmonary hypertension in case 5, the latter representing a plausible contributor to exercise intolerance. Case 9 had mild azotemia, but chronic kidney disease and IRIS staging could not be confirmed without urinalysis. Despite the chronic nature of the exercise intolerance and excessive panting, which had been ongoing for several months prior to presentation, the notable clinical improvement observed in all dogs following strictly gastrointestinal-directed medical and dietary therapies supports the hypothesis of a possible gastrointestinal contribution but does not establish causality. Clinical response to gastrointestinal-directed interventions was part of the inclusion criteria, thereby introducing selection and incorporation bias. Accordingly, treatment response cannot be considered diagnostic confirmation of gastrointestinal disease. Therapeutic interventions were also heterogeneous and frequently combined, precluding assessment of the individual effects of dietary modification, gastrointestinal-directed medications, or cobalamin supplementation.

Finally, the assessment of clinical improvements was subjective, relying primarily on owner perception. Accordingly, a caregiver placebo effect cannot be excluded, as outcomes were based on owner-reported assessments and no control group or objective measure of exercise tolerance (such as the 6-min walk test) was available. In human medicine, five validated scales exist to define and assess IBD-related fatigue, but no equivalent scales have been developed or validated in veterinary medicine to the authors’ knowledge (46). Although exercise intolerance can be objectively quantified in dogs via the 6-min walk test, this was not performed at the time of the investigations (47).

## 5 Conclusion

Exercise intolerance and excessive panting can originate from primary cardiac, respiratory, or extracardiac/extrarespiratory diseases. Once comprehensive diagnostic investigations render cardiac, respiratory, metabolic, endocrine, orthopedic, and neuromuscular etiologies unlikely, possible gastrointestinal involvement may be considered, even in the absence of overt digestive signs. Subtle ancillary signs reflecting visceral or abdominal discomfort should be actively investigated, and serum folate and cobalamin measurements should be considered during the diagnostic workup. Ultimately, empirical therapeutic trials consisting of gastrointestinal-directed medical treatment and dietary modifications may be considered, even though clinical improvement following such interventions cannot be interpreted as diagnostic confirmation.

This case series introduces the hypothesis that exercise intolerance and excessive panting may be associated with gastrointestinal disease in dogs, potentially mirroring IBD-related fatigue syndromes in human medicine.

Our study was not designed to explain the mechanisms leading to these clinical signs (e.g., delayed gastric emptying or intestinal dysbiosis for instance); further studies are warranted to confirm our findings and elucidate the underlying pathophysiological mechanisms in dogs.

## Data Availability

The original contributions presented in the study are included in the article/Supplementary material, further inquiries can be directed to the corresponding author.
