# Supplementary material for: Beyond the lungs and heart: a possible association between digestive disease and exercise intolerance in 10 dogs
Source: Front Vet Sci. 2026 Oct 28;13:1915931. doi: 10.3389/fvets.2026.1915931 (PMC13751425; doi:10.3389/fvets.2026.1915931)
Supplement: Supplementary file 1 [file Table_1.DOCX]

**Table S1. Selected physical examination findings at initial visit for each case enrolled in the study**

| **Parameters** | Case 1 | Case 2 | Case 3 | Case 4 | Case 5 | Case 6 | Case 7 | Case 8 | Case 9 | Case 10 |
| --- | --- | --- | --- | --- | --- | --- | --- | --- | --- | --- |
| Rectal temperature (°C) | 39.0 | 38.1 | 38.4 | 38.2 | 39.0 | 39.1 | 38.4 | 38.3 | 38.9 | 38.2 |
| Heart rate (bpm) | 64 | 80 | 100 | 108 | 88 | 100 | 120 | 80 | 66 | 80 |
| Heart murmur | No | No | RBDM (1/6) | No | No | No | No | LASM (4/6) | No | No |
| Cardiac rhythm | Sinus arrhythmia | Sinus arrhythmia | Sinus arrhythmia | Regular | Regular | Regular | Regular | Sinus arrhythmia | Regular | Regular |
| Respiratory rate (bpm) | 36 | 40 | 32 | 60 | 80 | >100 | 40 | 28 | >100 | 24 |
| Abdominal discomfort | Mild | No | Moderate | No | No | No | No | Moderate | Mild | Mild |
| Other findings | None | Stomatitis | Thickened intestinal wall | None | None | None | None | Stomatitis, mild mandibular lymphadenopathy, mild tail alopecia, eructation | Flatulence, papules and crusts on the chest and thoracic limbs, left prescapular lymphadenopathy | No |

LASM: Left apical systolic murmur; RBDM: Right basal diastolic murmur
