# Supplementary material for: Beyond the lungs and heart: a possible association between digestive disease and exercise intolerance in 10 dogs
Source: Front Vet Sci. 2026 Oct 28;13:1915931. doi: 10.3389/fvets.2026.1915931 (PMC13751425; doi:10.3389/fvets.2026.1915931)
Supplement: Supplementary file 2 [file Table_2.DOCX]

**Table S2a. Summary of diagnostic tests performed in the study population**

| **Test** | **Number of dogs** |
| --- | --- |
| Biochemistry | 10 |
| Complete blood count | 10 |
| Serum folates and cobalamin concentration | 10 |
| Electrolytes | 8 |
| Plasma ionized calcium concentration | 8 |
| Serum CRP concentration | 8 |
| Venous blood gas analysis | 8 |
| Thoracic radiographs | 7 |
| Echocardiography | 6 |
| Abdominal ultrasound | 5 |
| Electrocardiogram | 5 |
| Serum total T4 concentration | 5 |
| Serum cTSH concentration | 5 |
| Arterial blood gas analysis | 4 |
| Plasma creatine kinase concentration | 3 |
| Serum resting cortisol concentration | 3 |
| ACTH stimulation test | 2 |
| Serum cholesterol and triglycerides concentration | 3 |
| Measurement of left atrium/aorta ratio | 2 |
| CT angiography | 1 |
| Bronchoscopy and bronchoalveolar lavage | 1 |
| Bile acid assay | 1 |
| DGGR pancreatic lipase activity | 1 |
| Baermann fecal analysis | 1 |

ACTH, adrenocorticotropic hormone; CRP, C-reactive protein; CT, computed tomography; cTSH, canine thyroid-stimulating hormone

**Table S2b. Diagnostic tests performed for each case enrolled in the study.**

| **Case** | **Diagnostic tests performed** |
| --- | --- |
| Case 1 | CBC; Biochemistry; CRP; B9/B12; Electrolytes; Ionized calcium; Arterial blood gas; CK; Serum bile acid fasting and post-prandial; Thoracic radiographs; Echocardiography; ECG (+Holter); Abdominal ultrasound |
| Case 2 | CBC; Biochemistry; CRP; B9/B12; Electrolytes; Ionized calcium; Venous blood gas; Thoracic radiographs; Abdominal ultrasound |
| Case 3 | CBC*; Biochemistry*; B9/B12; Total proteins; Urea; Creatinine; Cholesterol; Triglycerides; DGGR lipase; Arterial blood gas; Thoracic radiographs; Echocardiography; ECG; Abdominal ultrasound |
| Case 4 | CBC; Biochemistry; CRP; B9/B12; Electrolytes; Ionized calcium; Venous blood gas; Total T4; cTSH; ACTH-stimulation test; Arterial blood gas; Thoracic radiographs; Echocardiography; ECG; Abdominal ultrasound |
| Case 5 | CBC*; Biochemistry*; B9/B12; cTLI; Thoracic radiographs; Echocardiography; Fecal examination (Baermann method); CT angiography; Bronchoscopy; BAL cytology, bile acid concentration, PCR Mycoplasma ssp. |
| Case 6 | CBC; Biochemistry; CRP; B9/B12; Electrolytes; Ionized calcium; Venous blood gas; Total T4; cTSH; Arterial blood gas; Thoracic radiographs; Echocardiography |
| Case 7 | CBC; Biochemistry; CRP; B9/B12; Electrolytes; Ionized calcium; Venous blood gas; Total T4; cTSH; CK; Arterial blood gas; Thoracic radiographs; LA/Ao value; ECG |
| Case 8 | CBC; Biochemistry*; CRP; B9/B12; Electrolytes; Ionized calcium; Venous blood gas; ACTH-stimulation test; Total T4; cTSH |
| Case 9 | CBC; Biochemistry; CRP; B9/B12; Electrolytes; Ionized calcium; Venous blood gas; LA/Ao value |
| Case 10 | CBC; Biochemistry; CRP; B9/B12; Electrolytes; Ionized calcium; Venous blood gas; CK; Total T4; cTSH; Resting cortisol; Echocardiography; UA; ECG; Abdominal ultrasound |

* performed by the referring veterinarian, reviewed at presentation, raw data not available

ACTH, adrenocorticotropic hormone; B9, serum folate concentration; B12, serum cobalamin concentration; BAL, bronchoalveolar lavage; BCS, body condition score; CBC, complete blood count; CK, plasma creatine kinase concentration; CRP, serum C-reactive protein concentration; CT, computed tomography; cTLI, canine trypsin-like immunoreactivity; cTSH, canine thyroid-stimulating hormone; E, entire; ECG, electrocardiogram; F, female; I, intact; LA/Ao, left atrium/aorta ratio; M, male; N, neutered; UA, urinalysis
