# Supplementary material for: Beyond the lungs and heart: a possible association between digestive disease and exercise intolerance in 10 dogs
Source: Front Vet Sci. 2026 Oct 28;13:1915931. doi: 10.3389/fvets.2026.1915931 (PMC13751425; doi:10.3389/fvets.2026.1915931)
Supplement: Supplementary file 3 [file Table_3.DOCX]

**Table S3. Selected bloodwork results for each case enrolled in the study**

| **Parameters** | Case 1 | Case 2 | Case 3 | Case 4 | Case 5 | Case 6 | Case 7 | Case 8 | Case 9 | Case 10 |
| --- | --- | --- | --- | --- | --- | --- | --- | --- | --- | --- |
| **CBC results** | | | | | | | | | | |
| RBC (x 10^12/L) | 8.0 | 5.5 | N | 6.4 | 7.4 | 7.1 | 6.3 | 6.0 | 6.8 | 6.1 |
| Hemoglobin (g/dL) | 17.5 | 12.0 | N | 15.1 | 17.3 | 17.0 | 16.0 | 14.4 | 12.8 | 14.0 |
| Hematocrit (%) | 54 | 36 | N | 42 | 51 | 49 | 41 | 38 | 37 | 40 |
| MCV (fL) | 68 | 65 | N | 66 | 69 | 69 | 65 | 63 | 55 | 66 |
| MCHC (g/dL) | 32 | 33 | N | 36 | 34 | 35 | 39 | 38 | 34.6 | 35 |
| MCH (pg) | 22 | 22 | N | 24 | 23 | 24 | 25 | 24 | 19 | 23 |
| WBC (x 10^9/L) | 5.6 | 6.0 | N | 8.7 | 12 | 14.4 | 6.3 | 10.8 | 7.9 | 9.3 |
| Neutrophils (x10^9/L) | 3.02 | 4.26 | N | 5.39 | 6.24 | 8.64 | 4.10 | 6.80 | 4.34 | 5.86 |
| Eosinophils (x 10^9/L) | 0.11 | 0.12 | N | 0.52 | 0.6 | 0.72 | 0.19 | 0.54 | 0.48 | 0.52 |
| Basophils (x 10^9/L) | 0 | 0 | N | 0 | 0 | 0 | 0 | 0 | 0 | 0 |
| Lymphocytes (x 10^9/L) | 2.30 | 1.20 | N | 2.09 | 4.44 | 4.46 | 1.70 | 2.81 | 2.76 | 1.97 |
| Monocytes (x 10^9/L) | 0.17 | 0.42 | N | 0.70 | 0.72 | 0.58 | 0.32 | 0.65 | 0.33 | 0.97 |
| Platelets (x 10^9/L) | 232 | 365 | N | 446 | 213 | 300 | 124 | 201 | 50 | 146 |
| Reticulocytes (x 10^9/L) | 80.8 | 80.3 | N | 19.2 | 80.7 | 94.5 | 102.6 | 55.8 | 8.8 | 40.7 |
| **Biochemistry profile results** | | | | |  |  |  |  |  |  |
| Urea (g/L) | 0.400 | 0.360 | 0.360 | 0.360 | 0.560 | 0.440 | 0.520 | 0.257 | 0.595 | 0.179 |
| Creatinine (mg/L) | 11 | 11 | 10 | 10 | 9 | 13 | 13 | 5.1 | 17 | 9 |
| Total proteins (g/L) | 63 | 69 | 69 | 62 | 60 | 66 | 66 | 64 | 63 | 68 |
| Albumin (g/L) | 37 | 36 | 33 | 35 | - | 37 | 31 | 25 | 31 | 32 |
| ALT (U/L) | 53 | 50 | 32 | 148 | 50 | 49 | 102 | 56 | 65 | 19 |
| ALP (U/L) | 17 | 58 | 10 | 30 | 101 | 51 | 26 | 28 | 37 | 194 |
| Glucose (g/L) | 0.89 | 0.92 | 0.95 | 1.18 | 0.99 | 1.08 | 0.88 | 0.98 | 0.86 | 0.95 |
| CRP (mg/L) | 3 | 7 | - | 1 | - | 7 | 31 | 7 | 7 | 14 |
| Sodium (mmol/L) | 153 | 147 | - | 146 | - | 149 | 141 | 143 | 143 | 144 |
| Potassium (mmol/L) | 3.7 | 4.2 | - | 3.8 | - | 3.8 | 4.5 | 4.5 | 4.2 | 4 |
| Chloride (mmol/L) | 112 | 113 | - | 119 | - | 105 | 118 | 116 | 114 | 117 |
| Ionized calcium (mmol/L) | 1.30 | 1.30 | - | 1.24 | - | 1.35 | 1.33 | 1.18 | 1.28 | 1.34 |
| Folates (ng/mL) | 11 | 19 | 12 | 8 | 4.1 | 16 | 8.7 | 14 | 23.1 | 7.53 |
| Cobalamin (ng/L) | 1017 | 750 | 241 | 348 | 150 | 326 | 1400 | 793 | 611 | 343 |
| Total T4 (nmol/L) | - | - | - | 2.1 | - | 19.4 | 33 | 40 | - | 15.2 |
| cTSH (ng/mL) | - | - | - | 0.12 | - | 0.05 | 0.20 | 0.07 | - | 0.13 |
| Creatine kinase (U/L) | 89 | - | - | - | - | - | 167 | - | - | 160 |
| Resting cortisol (nmol/L) | - | - | - | 96 | - | - | - | 39 | - | 107 |
| Post-ACTH cortisol (nmol/L) | - | - | - | 339 | - | - | - | 177 | - | - |
| Cholesterol (g/L) | - | - | 2.17 | - | - | - | 1.19 | - | - | 2.98 |
| Triglycerides (g/L) | - | - | 0.50 | - | - | - | 0.90 | - | - | 0.67 |
| Fasting serum bile acids (µg/L) | 27.1 | - | - | - | - | - | - | - | - | - |
| Postprandial serum bile acids (µg/L) | 25.9 | - | - | - | - | - | - | - | - | - |
| cTLI (µmol/L) | - | - | - | - | 9 | - | - | - | - | - |

N: Normal according to the medical record but raw values not available
