# Supplementary material for: Beyond the lungs and heart: a possible association between digestive disease and exercise intolerance in 10 dogs
Source: Front Vet Sci. 2026 Oct 28;13:1915931. doi: 10.3389/fvets.2026.1915931 (PMC13751425; doi:10.3389/fvets.2026.1915931)
Supplement: Supplementary file 4 [file Table_4.DOCX]

**Table S4. Individual findings supporting suspected gastrointestinal involvement and diagnostic interpretation**

| **Case** | **Potentially relevant findings** | **Gastrointestinal investigations** | **Diagnostic interpretation** |
| --- | --- | --- | --- |
| **1** | Low BCS (3/9); mild abdominal discomfort; increased cobalamin concentration (1017 ng/L); normal folate concentration. | Abdominal ultrasonography: no significant abnormalities. | No objective evidence of gastrointestinal disease. Gastrointestinal involvement suspected from nonspecific clinical findings and response to gastrointestinal-targeted therapeutic and alimentary measures; no definitive diagnosis based on histology. |
| **2** | Excessive grass eating, yawning, and air licking; increased folate concentration (19 ng/L); normal cobalamin concentration. | Abdominal ultrasonography: mild gastroenteropathy and findings suggestive of chronic pancreatitis. | Ultrasonographic findings suggestive of mild gastroenteropathy and chronic pancreatitis; no definitive diagnosis based on histology. |
| **3** | Excessive grass eating; moderate abdominal discomfort; cobalamin concentration <250 ng/L; normal folate concentration. | Abdominal ultrasonography: no significant abnormalities. | Suspected gastrointestinal involvement based on clinical findings and low cobalamin concentration; no definitive diagnosis based on histology. |
| **4** | Cobalamin insufficiency (348 ng/L); normal folate concentration. | Abdominal ultrasonography: mildly hyperechoic liver and biliary sludge, without relevant gastrointestinal abnormalities. | Suspected gastrointestinal involvement based primarily on cobalamin insufficiency; no definitive diagnosis based on histology. |
| **5** | Low BCS (3/9); cobalamin deficiency (150 ng/L); decreased folate concentration (4.1 ng/L). | Mild erythema of the arytenoid and epiglottic mucosa; bile acids detected in BAL fluid (1.1 µmol/L in LB1); no direct assessment of gastroesophageal reflux or gastrointestinal motility. | Possible gastrointestinal and aerodigestive involvement; gastroesophageal reflux confirmed by detection of bile acids in the BAL fluid. No definitive diagnosis of the gastrointestinal disease based on histology |
| **6** | Cobalamin insufficiency (326 ng/L); normal folate concentration. | No abdominal ultrasonography, endoscopy, histology, or gastrointestinal function testing. | Suspected gastrointestinal involvement based primarily on cobalamin insufficiency and response to gastrointestinal-targeted therapeutic and alimentary measures; no definitive diagnosis based on histology. |
| **7** | No gastrointestinal clinical signs or abdominal discomfort; increased cobalamin concentration (>1400 ng/L). | No abdominal ultrasonography, endoscopy, histopathology, or gastrointestinal function testing. | No objective evidence of gastrointestinal disease. Gastrointestinal involvement suspected from response to gastrointestinal-targeted therapeutic and alimentary measures; no definitive diagnosis based on histology. |
| **8** | Throat-clearing, excessive grass eating, previous diarrhea, and moderate abdominal discomfort; normal cobalamin and folate concentrations. | No abdominal ultrasonography, endoscopy, histopathology, or gastrointestinal function testing. | Suspected gastrointestinal involvement based on clinical findings and response to gastrointestinal-targeted therapeutic and alimentary measures; no definitive diagnosis based on histology. |
| **9** | Throat-clearing, flatulence, eructation, hyporexia, low BCS (3/9), mild abdominal discomfort, and increased folate concentration (23.1 ng/L); normal cobalamin concentration. | Abdominal ultrasonography declined; no direct assessment of dysbiosis. | Suspected gastrointestinal involvement based on clinical and laboratory findings; no definitive diagnosis based on histology. |
| **10** | Throat-clearing, flatulence, mild abdominal discomfort, and cobalamin insufficiency (343 ng/L); normal folate concentration. | Abdominal ultrasonography: no significant abnormalities. | Suspected gastrointestinal involvement based on clinical and laboratory findings; no definitive diagnosis based on histology. |

Abbreviations: BAL, bronchoalveolar lavage; BCS, body condition score; LB1, left cranial lobar bronchus.
