# Supplementary material for: Beyond the lungs and heart: a possible association between digestive disease and exercise intolerance in 10 dogs
Source: Front Vet Sci. 2026 Oct 28;13:1915931. doi: 10.3389/fvets.2026.1915931 (PMC13751425; doi:10.3389/fvets.2026.1915931)
Supplement: Supplementary file 5 [file Table_5.DOCX]

**Table S5. Individual cobalamin concentrations, supplementation protocols, and timing of clinical improvement in dogs with cobalamin insufficiency or deficiency**

| **Case** | **Baseline cobalamin concentration (ng/L)** | **Dose and route** | **Duration** | **Post-supplementation cobalamin concentration** | **Timing of reassessment** | **Time to complete clinical resolution (days)** |
| --- | --- | --- | --- | --- | --- | --- |
| 3 | 251 | No supplementation | NA | NA | 24 days (326 ng/L) | 24 |
| 4 | 348 | No supplementation | NA | NA | 174 days (244 ng/L; a supplementation was prescribed despite clinical resolution) | 170 |
| 5 | <150 | 1 mg orally once daily (2 capsules) | 3 months | Not assessed at our hospital | NA | 21 |
| 6 | 326 | No supplementation | NA | NA | NA | 30 |
| 10 | 343 | 1 mg orally once daily (2 capsules) | 3 months | Not assessed at our hospital | NA | 8 |

**Notes:** All dogs received Cobalaplex® (ADM Protexin Ltd., Somerset, UK). Each capsule contained 0.5 mg of cyanocobalamin and 0.2 mg of folic acid, together with a prebiotic formulation. All five dogs weighed >20 kg and received two capsules daily. Exact numerical post-supplementation cobalamin concentrations were unavailable; medical records indicated that concentrations were within the laboratory reference interval in all five dogs. No measurements were performed between treatment initiation and post-supplementation reassessment; therefore, the precise timing of cobalamin normalization relative to clinical improvement could not be determined.

Abbreviation: RI, reference interval.
