# Supplementary material for: Beyond the lungs and heart: a possible association between digestive disease and exercise intolerance in 10 dogs
Source: Front Vet Sci. 2026 Oct 28;13:1915931. doi: 10.3389/fvets.2026.1915931 (PMC13751425; doi:10.3389/fvets.2026.1915931)
Supplement: Supplementary file 6 [file Table_6.DOCX]

**Table S6. Cytological analysis of the BAL performed in case 5.**

|  | **Right cranial lobar bronchus (RB1)** | **Left cranial lobar bronchus (LB1)** | **Reference range** (17,43) |
| --- | --- | --- | --- |
| **Total cell count (cells/µL)** | 220 | 240 | < 500 |
| **% of macrophages** | 74 | 77 |  |
| **% of neutrophils** | 7 | 9 | < 12 |
| **% of eosinophils** | 12 | 7 | < 14 |
| **% of lymphocytes** | 7 | 7 | < 16 |
| **Bile acid concentration**  (µmol/L) | 0.1 | 1.1 | < 0.1 |
